# **Progression of peritoneal adenomucinosis to the scrotum: a rare occurrence treated with cytoreductive surgery and hyperthermic chemoperfusion of the scrotum in two patients**.

**Published:** 2014-06-30

**Authors:** Armando Sardi, William Andrés Jiménez, Chukwuemeka Wosu

**Affiliations:** Division of Surgical Oncology. The Institute for Cancer Care - Mercy Medical Center. Baltimore, Maryland, USA.

**Keywords:** Hyperthermic chemoperfusion, scrotum, DPAM, cytoreductive surgery, CRS, HIPEC

## Abstract

**Introduction:**

Disseminated Peritoneal Adenomucinosis (DPAM) is an infrequent presentation of appendiceal cancer. Infrequently, umbilical or inguinal hernias could be the first clinical manifestation of this condition; DPAM extension to the scrotum may be anatomically viable. Treatment with cytoreductive surgery (CRS) and hyperthermic intraperitoneal chemotherapy (HIPEC) is the standard of treatment for DPAM. We hypothesize that these same treatment principles, consisting of CRS with hyperthermic chemoperfusion of the scrotum (HCS), could be applied to the scrotal dissemination of DPAM.

**Methods:**

We reviewed our Institution's prospective cancer database and identified two cases of DPAM with extension to the scrotum. Their medical records were examined, and close follow-up was performed. Tumor histopathology and cytoreduction scores were evaluated. Tumor progression was monitored on follow-up by physical examination, tumor markers (CEA, CA 125, CA 19.9) and abdomino-pelvic CT scan.

**Results:**

Two patients who previously had CRS/ HIPEC for DPAM were successfully treated with HSC. Both patients are alive and free of disease at 88 and 57 months following initial CRS/HIPEC, and 50 and 32 months following CRS/HCS, respectively.

**Conclusion:**

Increased awareness by surgeons to the coexistence of inguinal hernia with peritoneal neoplasm and the need for a surgical repair is raised. CRS/HCS may be employed to treat patients with DPAM extension to the scrotum. Successful outcome is dependent on complete cytoreduction of metastatic tumor.

## Abstract (es)

**Introducción:**

La Adenomucinosis Peritoneal Diseminada (DPAM, por el término en inglés) es una presentación no frecuente del cáncer de apéndice. Infrecuentemente, las hernias umbilicales o inguinales pueden ser la primera manifestación clínica de esta condición; la extensión al escroto puede ser anatómicamente viable. La cirugía citoreductiva (CRS, por el término en inglés) con quimioterapia hipertérmica intraperitoneal (HIPEC, por el término en inglés) es el tratamiento estándar para DPAM. Nuestra hipótesis es que los mismos principios terapéuticos, consistentes en CRS con quimioterapia hipertérmica del escroto (HCS), pueden ser aplicados para DPAM con extensión al escroto.

**Métodos:**

Revisamos una base de datos prospectiva en nuestra Institución donde se identificaron dos casos de DPAM con extensión al escroto. Se examinaron sus historias clínicas, y se realizaron controles cercanos. La histopatología tumoral y la citoreducción fueron evaluados. La progresión tumoral fue monitorizada en los controles mediante examen físico, marcadores tumorales (CEA, CA 125, CA 19.9) y TAC abdomino-pélvico.

**Resultados:**

Dos pacientes a quienes se les practicó previamente CRS/HIPEC por DPAM fueron exitosamente tratados con CRS/HSC. Ambos pacientes se encuentran vivos y sin evidencia de enfermedad 88 y 57 meses después de la CRS/HIPEC inicial y a 50 y 32 meses post CRS/HCS, respectivamente.

**Conclusión:**

La precaución de los cirujanos sobre la coexistencia de hernias inguinales con neoplasias peritoneales y la necesidad de reparo quirúrgico debe ser incrementada. La CRS/HCS puede ser empleada en el tratamiento a pacientes con DPAM con extensión al escroto. Los resultados clínicos son dependientes de una citoreducción completa del tumor metastásico.

## Introduction

Disseminated peritoneal adenomucinosis (DPAM) is a common presentation of tumors arising from the appendix, it is frequently associated with extensive peritoneal involvement 1 ^,^ 2. Its mucinous overproduction could trigger unspecific symptoms, challenging appropriate diagnosis. Initial clinical presentation may be characterized by only an inguinal and/or umbilical hernia filled with mucin. However, no clear diagnosis or treatment option has been proposed for the scrotal extension of DPAM. 

Cytoreductive surgery (CRS) followed by hyperthermic intraperitoneal chemotherapy (HIPEC) has been recommended as a standard of care for patients with DPAM 2 ^-^ 4, increasing long term survival up to 80% and 75% at 5 and 10 years, respectively 5 ^-^ 7. We hypothesize that the involvement of a hernia sac, given that it is an extension of the peritoneum, could be treated in a similar manner. We report two cases of DPAM with scrotal recurrence following a successful CRS/HIPEC. The scrotal recurrence was treated with inguinal CRS and orchiectomy followed by hyperthermic chemoperfusion of the scrotum (HCS). 

## Hyperthermic chemoperfusion of the scrotum

### Technique:

**Figure 1.**
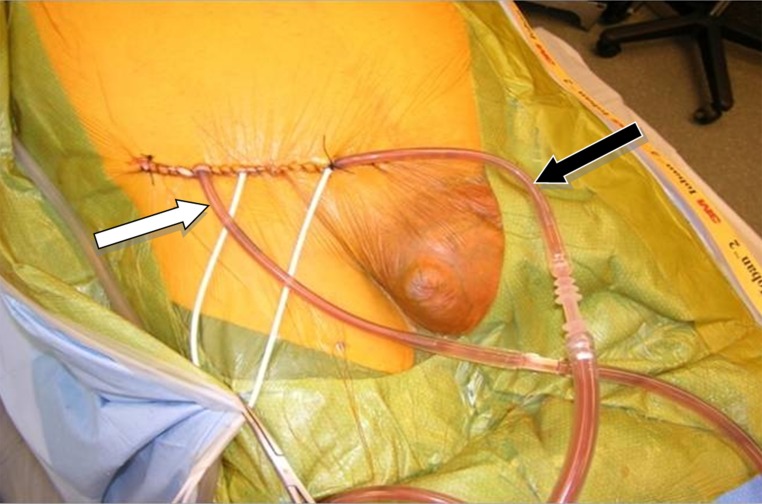
Hyperthermic chemoperfusion of the scrotum (HCS). Figure showing external view of catheter placement during hyperthermic chemoperfusion to the scrotum at the right groin. Black arrow: Inflow catheter extending to the scrotum. White arrow: Outflow catheter extending to the proximal groin.

Upon completion of CRS, two catheters (an inflow and outflow) were inserted into the groin. These catheters were secured temporarily with sutures (Fig. 1). While the inflow catheter extends into the scrotum, the outflow catheter remained proximal in the groin. Hyperthermic chemoperfusion of the scrotum was carried out by using 20 mg of Mitomycin-C for 60 min with an inflow temperature maintained at 43° C and an outflow temperature of 41-42° C.

## Case #1

**Figure 2.**
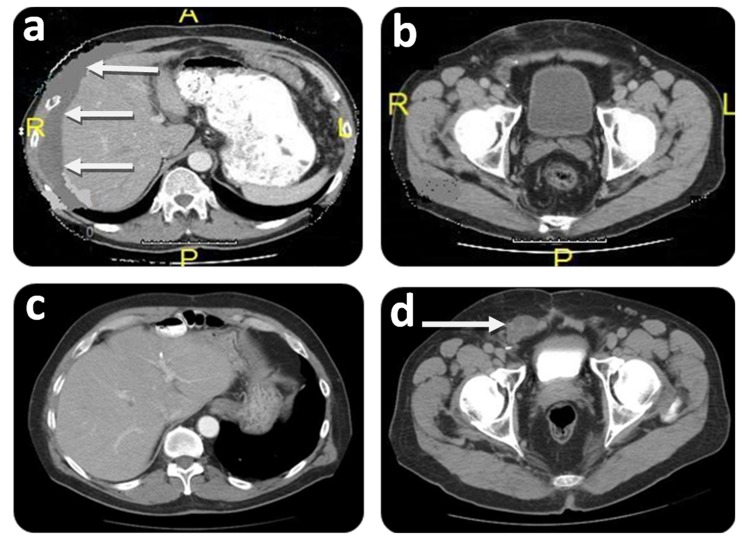
Initial and follow-up CT scan findings of Case # 1. Preoperative: **A)** CT scan shows intraperitoneal mucin around liver (white arrows) at initial CRS/HIPEC. A: Anterior; P: Posterior; R: Right side; L: Left side. **B)** CT scan shows no groin involvement at initial CRS/HIPEC. P: Posterior; R: Right side; L: Left side. Postoperative (36 months after CRS/HIPEC): **C)** CT scan shows no evidence of disease within the peritoneal cavity at time of groin recurrence. **D**) CT scan showing groin mass recurrence extending into the scrotum (white arrow).

A 65-year-old male presented with an inguinal and incarcerated umbilical hernia. At the time of surgery, he had mucinous collection in the umbilical hernia sac. The procedure was converted to a laparotomy with an appendectomy and peritoneal biopsy. He required umbilical and inguinal herniorrhaphy, requiring mesh for the latter after excision of the hernia sac. Pathology revealed that the tissue specimen was consistent with mucinous cystadenoma of the appendix with pseudomyxoma peritonei - DPAM. A CT scan revealed multiple areas of mucinous collection around the liver and omental involvement of the tumor (Fig. 2A).

Two months later, the patient was referred to our center specializing on peritoneal surface malignancy. Following a detailed history, the groin was carefully examined and hernia recurrence was ruled out. The CT scan showed diffuse peritoneal implants. No tumor was seen in the groin (Fig. 2B). The patient underwent a CRS/HIPEC (closed technique) using 40 mg of Mitomycin-C for 90 min (30 mg given at time 0 and 10 mg 30 min later) with an inflow temperature maintained at 43° C and an outflow temperature of 41-42° C. The pre/post surgical peritoneal carcinomatosis index (PCI) was 32/0, and complete cytoreduction (CC-0) was achieved. On post operative day 7, the patient developed pneumonia successfully treated with antibiotics and was discharged on post operative day 21 without further complications. He underwent close follow-up, and 36 months after CRS/HIPEC, patient complained of right scrotal edema and a right groin discomfort. No evidence of intra-abdominal disease was evident on CT scan (Fig. 2C); however, and a right groin mass was identified (Fig. 2D). Mucin was obtained with a fine needle aspiration of the scrotum. At the diagnostic laparoscopy, a groin recurrence with extension to the scrotum was strongly suspected and intra-abdominal recurrence was ruled out. Two months later the patient was subjected to exploration of the right groin with radical tumoral resection, resection of abdominal wall, orchiectomy and HCS (Fig. 1). Complete cytoreduction was achieved. The patient was discharged on post operative day 5 without complication. On close follow-up, the patient is alive and without evidence of disease (NED) 88 months from the first CRS/HIPEC, and 50 months from CRS/HCS.

## Case #2

A 63-year-old male presented with right lower abdominal pain. Appendicitis was suspected and his appendectomy was converted to an exploratory laparotomy due to incidental findings of ruptured tumor of the appendix and DPAM. The patient required a right hemicolectomy at that time. Pathology showed ruptured mucinous cystadenoma of the appendix with pseudomyxoma peritonei - DPAM. Eleven months later, a left inguinal hernia was diagnosed, and during the mesh herniorrhaphy peritoneal and hernial sac mucinous deposits were identified. 

**Figure 3.**
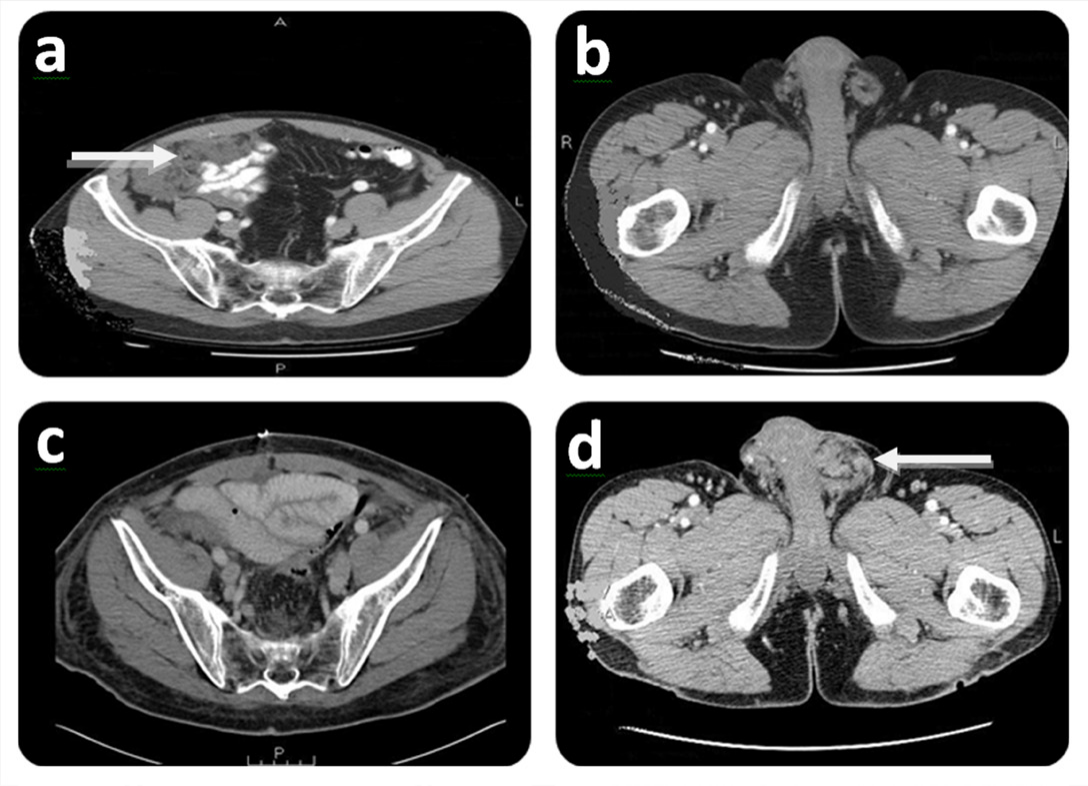
Initial and follow-up CT scan findings of Case # 2. Preoperative: **A)** CT scan shows right lower abdominal mass (white arrow) at initial CRS/HIPEC. A: Anterior; P: Posterior. **B)** CT scan shows no groin involvement at initial CRS/HIPEC. R: Right side; L: Left side. Postoperative (7 months after CRS/HIPEC): **C)** CT scan shows no evidence of disease at time of scrotal recurrence. P: Posterior. **D)** CT scan showing left scrotal mass (white arrow). L: Left side.

Five months after the herniorrhaphy the patient was referred to our Institution with a complaint of right lower abdominal discomfort. Further evaluation with a CT scan showed diffuse peritoneal metastases (Fig. 3A). No groin disease was found on physical examination or CT scan (Fig. 3B). Three weeks later, the patient underwent a CRS/HIPEC for DPAM. The pre/post surgical PCI was 36/0 and a CC-0 was achieved. Hyperthermic intraperitoneal chemotherapy (closed technique) was carried out by using 40 mg of Mitomycin-C for 90 min (30 mg given at time 0 and 10 mg 30 min later) with inflow temperature maintained at 43° C an outflow temperature of 41-42° C. Seven months later, on follow up, the patient presented with left groin discomfort and a scrotal mass. A CT scan identified no evidence of intra-abdominal disease (Fig. 3C), and a left scrotal mass (Fig. 3D). Fine-needle aspiration determined the existence of mucin in the left scrotum. Two months later the patient had CRS including left orchiectomy, removal of *in-situ* Prolene^®^ mesh, HCS (Fig. 1) and subsequent repair of inguinal defect with Prolene^®^ mesh. Complete cytoreduction was achieved at this stage. The patient was discharged on post-operative day 3 without complication. On follow up, the patient is considered NED at 57 months after his initial CRS/HIPEC and 32 months post CRS/HCS. 

## Discussion

Peritoneal dissemination is a common presentation of appendiceal tumors regardless of the grade of histopathology 4. An inguinal or umbilical hernia could be the first manifestation of this condition. Of historic interest, Sister Mary Joseph nodule is a palpable nodule bulging into the umbilicus as a result of metastases of a malignant cancer in the gastrointestinal tract or pelvis. Sister Mary Joseph Dempsey was the surgical assistant of William J Mayo. She drew Mayo's attention to this phenomenon and, in 1949, Hamilton Bailey coined the term after her. 

CRS/HIPEC is considered the standard of care for DPAM with complete cytoreduction being a key component 2 ^,^ 4. A prospective study of patients with DPAM treated with CRS/HIPEC has shown a five -and ten- year survival of 80% and 75%, respectively 5 ^-^ 7. Similarly, treatment with CRS/HIPEC has been shown to confer a survival advantage in patients with high grade appendiceal tumors 8 ^-^ 10. Because complete cytoreduction is the goal in treating patients with DPAM, it is essential to identify the presence of an undiagnosed patent processus vaginalis at the time of surgery, given that it may serve as a progression site. Therefore, hernia repair in the presence of DPAM may give rise to recurrence due to seeding of tumor cells at the time of repair. 

It is reasonable to suggest that extension of DPAM to the scrotum may be attributed to either an unrecognized indirect inguinal hernia at the time of the initial surgery or to inadequate exposure of the hernia sac to the chemotherapeutic agent at the time of HIPEC. If a hernia is present in a DPAM patient, it is likely to result in tumoral metastasis to the hernial sac. Therefore, ruling out the presence of hernias during the physical examination is extremely important. Furthermore, removal of the hernial sac is imperative during the surgical procedure.

We consider that the recurrence of mucinous neoplasm (DPAM) in the scrotum should be an indication for complete removal of all tumor and hyperthermic chemoperfusion. Herein, the same principles that are applied for intra-abdominal recurrence, like hernias, are a direct extension of the peritoneum.

In conclusion, increased awareness by surgeons of the coexistence of inguinal hernia with peritoneal neoplasm and the need for a surgical repair is raised. CRS and HCS may be employed in treating patients with DPAM extension to the scrotum. Successful outcome is dependent on complete cytoreduction of metastatic tumor. 
